# Ectomycorrhizal fungal communities of secondary tropical forests dominated by *Tristaniopsis* in Bangka Island, Indonesia

**DOI:** 10.1371/journal.pone.0221998

**Published:** 2019-09-09

**Authors:** Helbert, Maman Turjaman, Kazuhide Nara

**Affiliations:** 1 Department of Natural Environmental Studies, The University of Tokyo, Kashiwa, Chiba, Japan; 2 Forest Research and Development Centre (FRDC), Environment and Forestry Research, Development, Innovation Agency (FORDA), the Ministry of Environment and Forestry, Bogor, Indonesia; University of California Berkeley, UNITED STATES

## Abstract

In Southeast Asia, primary tropical rainforests are usually dominated by ectomycorrhizal (ECM) trees belonging to Dipterocarpaceae, although arbuscular mycorrhizal trees often outcompete them after disturbances such as forest fires and clear-cutting, thus preventing dipterocarp regeneration. In some secondary tropical forests, however, potentially ECM trees belonging to *Tristaniopsis* (Myrtaceae) become dominant and may help ECM dipterocarp forests to recover. However, we have no information about their mycorrhizal status in these settings. In this study, we analyzed ECM fungal communities in tropical secondary forests dominated by *Tristaniopsis* and investigated which ECM fungal species are shared with other tropical or temperate areas. In total, 100 samples were collected from four secondary forests dominated by *Tristaniopsis* on Bangka Island. ECM tips in the soil samples were subjected to molecular analyses to identify both ECM and host species. Based on a >97% ITS sequence similarity threshold, we identified 56 ECM fungal species dominated by Thelephoraceae, Russulaceae, and Clavulinaceae. Some of the ECM fungal species were shared between dominant *Tristaniopsis* and coexisting *Eucalyptus* or *Quercus* trees, including 5 common to ECM fungi recorded in a primary mixed dipterocarp forest at Lambir Hill, Malaysia. In contrast, no ECM fungal species were shared with other geographical regions, even with *Tristaniopsis* in New Caledonia. These results imply that secondary tropical forests dominated by *Tristaniopsis* harbor diverse ECM fungi, including those that inhabit primary dipterocarp forests in the same geographical region. They may function as refugia for ECM fungi, given that dipterocarp forests are disappearing quickly due to human activity.

## Introduction

Tropical forests account for approximately 44% of the world’s forest coverage[1]. However, from 2000 to 2010, there was a net forest loss of 7 million hectares per year in tropical countries, mainly due to large-scale commercial agriculture[1]. These dramatic losses and changes in tropical forests are becoming a serious threat to biodiversity, given that tropical rainforests sustain the largest number of species in the world[2].

Dominant trees in many forest ecosystems are associated with ectomycorrhizal (ECM) fungi and depend on them for soil nutrients, which they exchange for photosynthesis products[3,4]. Thus, ECM symbiosis is regarded as a prerequisite for host tree growth and survival in nature[5]. In fact, the availability of ECM fungi and their composition could be the most significant determinant of seedling establishment in heavily disturbed areas[6]. Therefore, ECM fungal communities have been studied repeatedly in disturbed areas under boreal[7], temperate[8], and subtropical climates[9]. However, no previous studies documented ECM fungal communities in heavily disturbed tropical areas.

Available data of ECM fungi in Southeast Asia are mostly from undisturbed Dipterocarpaceae forests[10–13], largely because this dominant ECM host is often replaced by fast growing arbuscular mycorrhizal trees, such as *Macaranga* and *Mallotus* after disturbance, [14,15]. However, in some parts of Southeast Asia[16,17], potentially ECM trees belonging to Myrtaceae become dominant in disturbed sites, although we know nothing about their ECM colonization and ECM fungal communities.

Myrtaceae includes both arbuscular mycorrhizal and ECM lineages. The latter includes *Eucalyptus*, on which ECM fungi have been documented in native areas like Australia[18] and in some areas to which they have been introduced, such as the Seychelles[19] and Africa[20]. *Tristaniopsis* is another ECM lineage in Myrtaceae, distributed in Cambodia, Myanmar, the Peninsula of Malaysia, Borneo, Java, Sumatra, and Australia[21]. Waseem et al. (2017) recently reported ECM fungi associated with endemic *Tristaniopsis* species in New Caledonia[22]. Some other *Tristaniopsis* species appear in secondary forests in Southeast Asia, where biogeographical, climate, and ecological conditions are fundamentally different from New Caledonia. We do not know whether *Tristaniopsis* trees are colonized by ECM fungi in these settings, and if colonized, what types of ECM fungal communities are present.

In this study, we investigated the ECM fungal communities of four secondary tropical forests dominated by *Tristaniopsis* on Bangka Island, Indonesia. Our objectives were (1) to confirm the ECM colonization of *Tristaniopsis* under secondary tropical forest settings, (2) to characterize ECM fungal diversity and species composition, and (3) to clarify how many fungal species are shared with known fungi on *Tristaniopsis* in New Caledonia and with fungi from other tropical or temperate areas. The knowledge obtained from this study will broaden our understanding of tropical ECM fungal ecology and biogeography, with implications for the regeneration of ECM forests in the tropics.

## Materials and methods

### Study sites

Bangka Island is located off the eastern coast of Sumatra Island, across the Bangka Strait. Four secondary forests with various disturbance types were selected: Kelapa (site 1), Limbung (site 2), Namang (site 3), and Sungai Selan (site 4), as shown in Table 1 and S1 Fig. All of these forests are dominated by *Tristaniopsis*, coexisting with other pioneer tree species. The sampling areas varied from 0.5 to 1.8 ha, depending on the remaining forest area. The average annual precipitation on this island is 2426 mm, most of which falls in the rainy season from July to December. The average monthly temperature on this island is 27.21 ± 0.43°C, representing a typical tropical climate with little seasonal temperature variation.

**Table 1:** Geographical and historical information of sampling sites.

Name	GPS	Site Area (ha)	Forest Established	Mean DBH* (cm)	Previous History
Kelapa (1)	S1°50’, E105°43’	1.5	2008	–	Paddy field
Limbung (2)	S1°44, E105°34’	1.8	2005	13.8±2.9	Clear cut & burn
Namang (3)	S2°22, E106°11’	1.2	2008	12.5±3.7	Selective cutting
Sungai Selan (4)	S2°26, E106°30’	0.73	1991	13.4±3.1	Clear cut & burn

*DBH: diameter at breast height of *Tristaniopsis* trees.

No specific permission was required to access all sampling locations. Kelapa. Limbung and Sungai Kelan are village forests which belong to the local people. We met the village heads, and they guided us to sampling location. Namang is an EcoEduTourism forest. Since this is educational forests, we didn't need any specific permission to access and do sampling activities. This field study did not involve any endangered or protected species.

### Soil sampling

Twenty-five soil samples (of dimensions 5 x 5 x 10 cm) were randomly collected from each sampling site in April 2015. Each sampling point was selected near a selected *Tristaniopsis* tree, which was >5 m away from other selected trees. The geographical positions of individual sampling points were recorded using GPS (GPSMAP62SJ, Garmin, Olathe, KS, USA). Soil samples were placed separately in plastic bags and stored at ambient temperature and processed within six days. Some leaves and flowers from *Tristaniopsis* trees were collected from the sampling sites to obtain reference DNA for host identification.

### Identification of ectomycorrhizal fungi

All ECM roots were carefully separated from each soil sample and cleaned in tap water. The cleaned ECM root tips were examined under a stereomicroscope (SZ61, Olympus Co., Tokyo, Japan) and grouped into morphotypes based on their morphological characteristics, including surface texture, mantle color, emanating hyphae, and rhizomorphs[23]. Whenever available, three ECM root tips were sampled from each morphotype per soil sample and placed into separate 2.0-ml tubes for DNA extraction. Morphotyping was completed within 6 days of soil sampling.

DNA extraction and molecular analysis were carried out following Murata et al. (2013), with minor modification[24]. Briefly, total DNA was extracted using the cetyltrimethyl ammonium bromide (CTAB) method. Internal transcribed spacer (ITS) regions (including ITS1, 5.8S, ITS2) of nuclear ribosomal DNA were amplified by polymerase chain reaction (PCR) with the ITS1F and ITS4 primers combination (S1 Table). For unsuccessfully amplified samples, other forward (ITS5 or ITS0FT) and reverse (LBW, LAW, or ITS4CG) primerswere used. A Platinum® Multiplex PCR Master Mix (Applied Biosystems, Foster City, CA, USA) was used for PCR. Successfully amplified PCR products were purified and subjected to Sanger sequencing reaction using BigDye Terminator Cycle Sequencing Kit (Applied Biosystem) with ITS1 as the sequence primer or ITS4 for poorly sequenced samples. Sequencing was performed on ABI 3730xl (Applied Biosystems).

All of the obtained sequences were verified against their original chromatograms, manually corrected, and grouped into molecular operational taxonomic units (hereafter referred to as ‘species’ for simplicity, following the practice in this field) based on sequence similarities greater than 97%, which we calculated using ATGC software (ver. 7.0; GENETYX Corp., Tokyo, Japan). Fungal identity was assigned based on BLAST search results against known taxa in the international sequence databases. Non-ECM sequence results were excluded from subsequent analysis.

The host associated with each ECM root tip was determined based on sequences in plastid *trn*L or *rbc*L regions of chloroplast DNA. Primers trnC (5′-cgaaatcggtagacgctacg-3′) or rbcla-F (5′-atgtcaccacaaacagagactaaagc-3′), in combination with trnD (5′-ggggatagagggacttgaac-3′) or rbcla-R (5′-gtaaaatcaagtccaccrcg-3′), were used for PCR amplification. All of the amplicons were purified and sequenced using trnC or rbcla-F as the sequencing primer. The sequences obtained from *Tristaniopsis* leaves were used as references.

### Statistical analysis

The frequency of each ECM species was defined as the number of soil samples containing that species. The species richness of ECM fungi at all sampling sites was estimated using EstimateS software ver. 9[25], and Jackknife2 with 1000 randomizations was used. The similarities between the ECM fungal communities of different sampling sites were visualized using a non-metric dimensional scaling (NMDS) procedure implemented in the ‘vegan’ package of R software ver. 3.3.1[26] with the Bray–Curtis distance and 999 permutations. The statistical differences between the ECM fungal community compositions of the sampling sites were evaluated using the Adonis (permutation multivariate analysis of variance) function of the ‘vegan’ package, using the Bray–Curtis distance and 999 permutations. The *Betadisper* test (Permutational analysis of multivariate dispersions) in ‘vegan’ was also used to determine the data dispersions of the communities between groups.

To evaluate how many ECM fungal species were shared with those found on *Tristaniopsis* in New Caledonia, our ITS sequences were compared with those in Waseem et al. (2017)[22]. If the similarity was >97% in a pairwise comparison between the two regions, we regarded it as the same species and as shared between the regions. To determine whether the fungal species found in this study had already been recorded in other geographical regions, a BLAST search was carried out against sequences deposited in the International Nucleotide Sequence Database (INSD). If BLAST matches showed similarities >97%, we derived their geographical positions from the metadata of the matched sequences.

## Results

Of the 100 soil samples collected from the four sites, 43 contained *Tristaniopsis* ECM roots. *Quercus* and *Eucalyptus* were confirmed in 4 and 3 soil samples, respectively. As a minor host, *Shorea* was detected only once. Thirty soil samples did not contain ECM root-tips. In terms of ECM root abundance, *Tristaniopsis* was dominant, accounting for 77.3% of ECM roots examined.

In total, we identified 56 ECM fungal species (accession number LC483889 –LC483944), of which 36 species were collected from *Tristaniopsis* roots (S2 Table). *Quercus* and *Eucalyptus* were associated with 4 ECM fungal species. Of the 36 species identified on *Tristaniopsis*, 2 species each were shared with *Quercus* and *Eucalyptus*. These shared species were found relatively frequently in the forests. An average of 1.77 (excluding null samples) ECM fungal species were detected per soil sample, with a maximum of 4 species.

**Fig 1:**
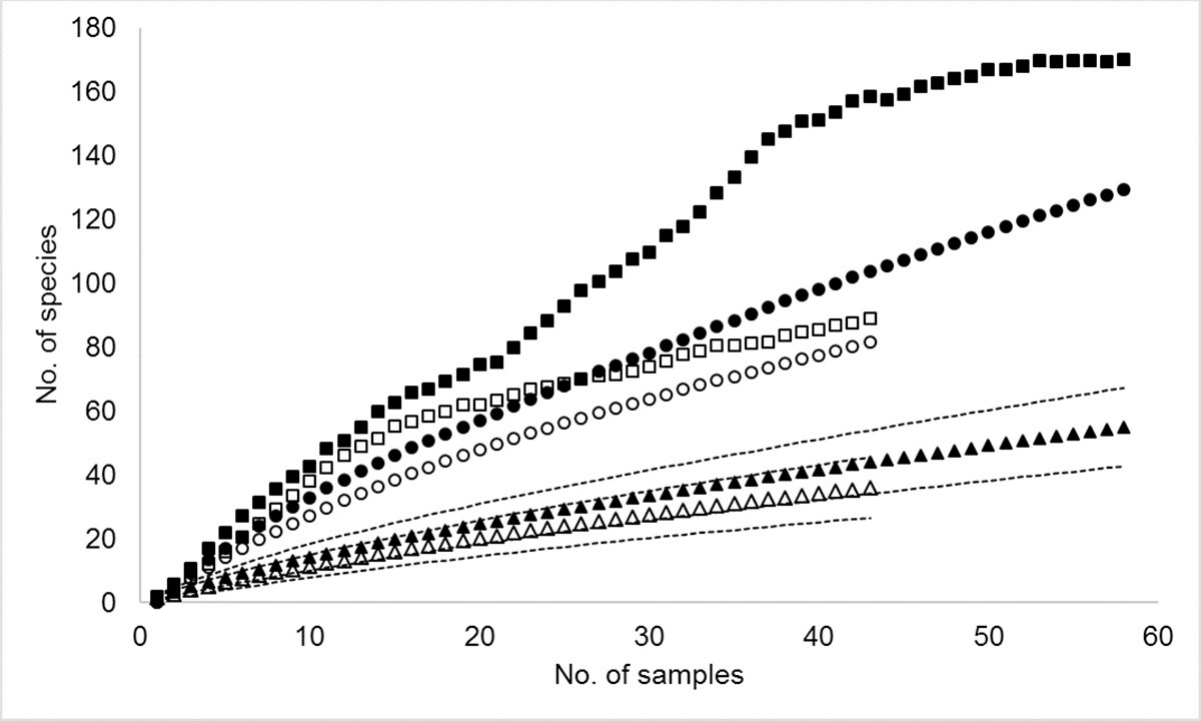
Species accumulation curves for ectomycorrhizal (ECM) fungi found in *Tristaniopsis* forests on Bangka Island. Filled and open triangles represent observed species richness of ECM fungi from all hosts and *Tristaniopsis*, respectively, with 95% confidence intervals. Jackknife2 (*circles*) and Chao2 (*squares*) minimum species richness estimates are shown for all host species and *Tristaniopsis* with filled and open symbols, respectively.

The Jack2 richness estimator indicated that at least 129 ECM fungal species would inhabit these forests, and for *Tristaniopsis* alone, 82 species were expected. The observed ECM fungal richness (and Jack2 estimates) at Kelapa, Limbung, Namang, and Sungai Kelan were 19 (41), 25 (60), 15 (36), and 9 (22), respectively. The species accumulation curves for all hosts and for *Tristaniopsis* did not approach the asymptote at our maximum sampling effort, indicating that additional species will be found with greater sampling effort (Fig 1). The diversity parameters are summarized in Table 2.

**Table 2:** Summary of ectomycorrhizal fungal diversity in secondary tropical forests dominated by *Tristaniopsis*.

Parameter	All sites	Site 1	Site 2	Site 3	Site 4
Soil core contain ECM	58	20	15	16	7
Observed richness	56	19	25	15	9
Mean richness per soil core	1.77	2.00	1.87	1.50	1.57
Jackknife2	129	41	60	36	22
Chao2	170	51	73	36	33
Shannon indices (H)	3.634	2.68	3.193	2.369	2.098
Simpson’s indices (D)	0.033	0.061	0.006	0.109	0.054

ECM fungal communities were dominated by a few species. Only 3 ECM fungal species were identified in more than 5 soil samples. In contrast, 42 species were singletons. The frequencies of *Cenococcum geophilum*, Thelephoraceae sp.1, and Thelephoraceae sp.2 were high on *Tristaniopsis* roots, found in 15, 7, and 6 soil samples, respectively. Thelephoraceae (11 spp.) was the most species-rich ECM fungal lineage, followed by Russulaceae (6 spp.), Clavulinaceae (5 spp.), and Amanitaceae (4 spp.).

ECM fungal communities were not separated by sampling location (*R*^*2*^ = 0.55, *P* = 0.227). A Mantel test revealed that the geographic distance did not affect the structure of ECM communities (*R* = -0.24, α = 0.89).

Most ECM fungi detected in this study shared no similar sequence (>97%) records in INSD and Unite, indicating newly confirmed species. Only 6 of the 56 ECM fungi had matches with deposited sequences at >97% similarities. Five of these (*Agaricomycetes* sp1, *Amanita* sp3, *Amanita* sp4, Boletaceae sp1, and Cortinariaceae sp1) are likely to be the same species as found at Lambir Hill National Park, Sarawak[12]. Another species matched the sequence of *Heimioporus* sp. sporocarp collected on Bangka Island (Accession no. KR061493). None of the ECM fungal species found in this study matched with sequences obtained from *Tristaniopsis* in New Caledonia[22]. Although numerous ECM fungal sequences are available, none of them had ITS similarity to our sequences of >97%, except for the above-mentioned records in Southeast Asia.

## Discussion

In this study, we found 56 ECM fungal species in 100 soil samples (58 soil samples containing ECM roots) collected from four *Tristaniopsis* forests. The observed species richness is far lower than that of temperate forests, after being rarefied to the same sampling effort[27]. ECM fungal richness is usually low in early successional stages, and increases with succession in temperate areas[8,28]. Although no previous studies have examined ECM fungal succession in the tropics, we may regard the lower richness as early successional ECM fungal communities affected by recent disturbance. Indeed, the diversity indices observed in this study were lower than other less-disturbed tropical forests documented in Borneo[12] and Thailand[13], where the Simpson indices were 0.038 and 0.053, respectively. The lower observed diversity may also be explained in part by the global pattern of ECM fungal diversity, as tropical areas have inherently less diverse ECM fungi[27].

The ECM fungal communities did not vary significantly between sampling sites. This does not indicate that ECM fungal communities are less affected by geographical positions and environmental conditions (e.g., temperature, rainfall, and elevation), but rather indicates that such effects would be inconspicuous at our sampling scale, as all of the studied sites were located on the same small island, with little climate variation. No ECM fungi were shared with *Tristaniopsis* in New Caledonia[22]. The geographic location and associated differences in environmental conditions may affect the ECM fungal composition, even associated with the same host genus, if we expand the geographical scale up to >6700 km, the distance between Bangka and New Caledonia. Bahram et al [29] showed tropical ECM fungal communities exhibited stronger distance-decay pattern.

There were no significant differences between the ECM fungal communities of co-existing host genera, partly because hosts other than *Tristaniopsis* were rare and were thus represented by fewer soil samples. However, two of the four ECM fungi found on *Eucalyptus* in this study were actually shared with *Tristaniopsis*, indicating their compatibility with both host genera. Because these two genera belong to the same family, Myrtaceae, they may be associated with similar ECM fungal communities in the same region. However, it would be premature to conclude this based on this study alone. It should also be noted that none of the *Eucalyptus* ECM fungi in other geographical regions matched our *Tristaniopsis* ECM fungi at the >97% sequence similarity threshold. In temperate areas, many ECM fungi were shared within the same host family, e.g. Pinaceae, across different continents[30], probably because of land bridges in ice ages. Biogeographical boundaries in the tropics (e.g., Wallace Line) remained disconnected even during ice ages[31,32], so the biogeographical effects of ECM fungal communities may be larger in the tropics than in temperate areas[29].

Five of the 56 ECM fungal species were recorded in a mixed dipterocarp forest at Lambir Hill, Sarawak, Malaysia[12], indicating their wide geographic distribution, with a range of at least 1100 km, but within the same biogeographical region, called Sundaland. Although the host species were not identified in Peay et al. (2010), it is likely that Dipterocarpaceae were the host, given the above-ground dominance of this tree group at Lambir Hill. Thus, the existence of the shared ECM fungi suggests their wide host range, including different host families. Indeed, two of the four ECM fungal species found on *Quercus* (Fagaceae) in this study were also found on *Tristaniopsis* (Myrtaceae). The ECM fungal sharing between the primary Lambir Hill forest and secondary *Tristaniopsis* forests implies that the pioneer *Tristaniopsis* trees could function as refugia for ECM fungi inhabiting dipterocarp forests. As ECM fungi on pioneer trees facilitate the establishment of successional ECM tree species in temperate regions[6], *Tristaniopsis* ECM fungi may help dipterocarp seedling establishment and forest recovery. It would be worth confirming this in future studies, given that dipterocarp forests have largely disappeared and there is currently no sign of recovery in many places in Southeast Asia.

## Conclusion

Secondary tropical rainforests dominated by *Tristaniopsis* trees in Bangka island were found to harbor diverse ECM fungi, many of which were new species that were not identified in previous studies. None of the ECM fungi were shared with *Tristaniopsis* in New Caledonia. In contrast, some ECM fungi were shared between *Tristaniopsis* and other coexisting tree genera at the study sites, and five species were common to those detected in primary dipterocarp forests in the same biogeographic region. While many tropical rainforests become arbuscular mycorrhizal ecosystems after disturbance, secondary forests dominated by *Tristaniopsis* trees remain ECM ecosystems. Hence, they could function as refugia for ECM fungi that inhabit primary mixed dipterocarp forests in Southeast Asia.

## Supporting information

S1 Fig Map of sampling site. (DOCX)

S1 Table PCR Primers used in this study. (DOCX)

S2 Table Ectomycorrhizal fungi; their frequency and host identity confirm in secondary *Tristaniopsis* forests, Bangka, Indonesia. (DOCX)
