# Supplementary figures and images for: Ectomycorrhizal fungal communities of secondary tropical forests dominated by Tristaniopsis in Bangka Island, Indonesia
Source: PLoS One. 2019 Sep 9;14(9):e0221998. doi: 10.1371/journal.pone.0221998 (PMC6733470; doi:10.1371/journal.pone.0221998)

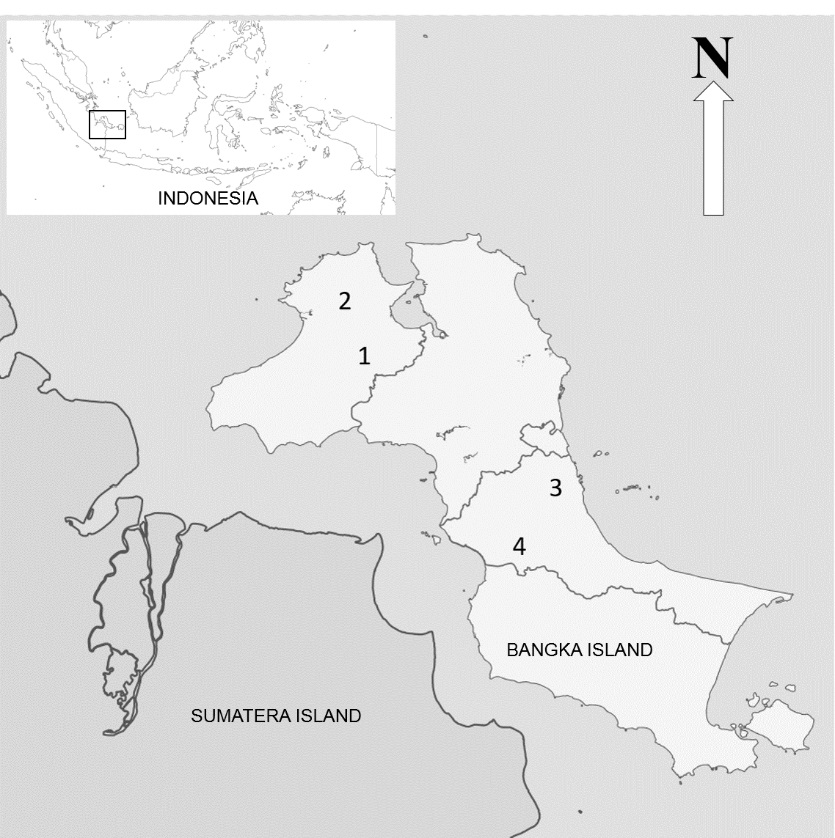


**S1 Figure. Map of sampling site.** 1) Kelapa; 2) Limbung; 3) Namang; 4) Sungai Kelan

Supplement: S1 Fig — (DOCX) [file pone.0221998.s001.docx]
