# Supplementary material for: Ectomycorrhizal fungal communities of secondary tropical forests dominated by Tristaniopsis in Bangka Island, Indonesia
Source: PLoS One. 2019 Sep 9;14(9):e0221998. doi: 10.1371/journal.pone.0221998 (PMC6733470; doi:10.1371/journal.pone.0221998)
Supplement: S1 Table — (DOCX) [file pone.0221998.s002.docx]

**S1 Table. PCR Primers used in this study**

| Primer name | Direction | Primer sequences (5’ – 3’) | Reference |
| --- | --- | --- | --- |
| ITS1F | Forward | CTTGGTCATTTAGAGGAAGTAA | Gardes & Bruns, (1993) |
| ITS4 | Reverse | TCCTCCGCTTATTGATATGC | White et al., (1990) |
| ITS5 | Forward | GGAAGTAAAAGTCGTAACAAGG | White et al., (1990) |
| ITS0FT | Forward | CTTGGTCATTTAGAGGAAGTAA | Tedersoo et al., (2006) |
| LBW | Reverse | CTTTTCATCTTTCCCTCACGG | Tedersoo et al., (2008) |
| LAW | Reverse | CTTTTCATCTTTCGATCACTC | Tedersoo et al., (2008) |
| ITS4CG | Reverse | CACATGGCAARGGCAACCG | Bahram et al., (2011) |

# **References**

Gardes M, Bruns TD. ITS primers with enhanced specificity for basidiomycetes - application to the identification of mycorrhizae and rusts. Mol Ecol. 1993;2(2): 113–118.

White TJ, Bruns T, Lee S, Taylor J. Amplification and direct sequencing of fungal ribosomal rna genes for phylogenetics. In: MA I, DH G, JJ S, TJ W, editors. PCR Protocols. United States: Elsevier; 1990 pp. 315–322.

Tedersoo L, Suvi T, Larsson E, Kõljalg U. Diversity and community structure of ectomycorrhizal fungi in a wooded meadow. Mycol Res. 2006;110(6): 734–748.

Tedersoo L, Jairus T, Horton BM, Abarenkov K, Suvi T, Saar I, et al. Strong host preference of ectomycorrhizal fungi in a Tasmanian wet sclerophyll forest as revealed by DNA barcoding and taxon-specific primers. New Phytol. 2008;180(2): 479–490. Available from: http://doi.wiley.com/10.1111/j.1469-8137.2008.02561.x

Bahram M, Põlme S, Kõljalg U, Tedersoo L. A single European aspen (*Populus tremula*) tree individual may potentially harbour dozens of *Cenococcum geophilum* ITS genotypes and hundreds of species of ectomycorrhizal fungi. FEMS Microbiol Ecol. 2011;75(2): 313–320.
