# Supplementary material for: Ectomycorrhizal fungal communities of secondary tropical forests dominated by Tristaniopsis in Bangka Island, Indonesia
Source: PLoS One. 2019 Sep 9;14(9):e0221998. doi: 10.1371/journal.pone.0221998 (PMC6733470; doi:10.1371/journal.pone.0221998)
Supplement: S2 Table — (DOCX) [file pone.0221998.s003.docx]

**S2 Table. Ectomycorrhizal fungi; their frequency and host identity confirm in secondary *Tristaniopsis* forests, Bangka, Indonesia**

| Species | Accession Number | Length | Best BLAST Match at NCBI | | UNITE species hypothesis number | Frequency | | | | HOST* |
| --- | --- | --- | --- | --- | --- | --- | --- | --- | --- | --- |
|  |  |  | Accession Number | Maximum Identity (%) |  | 1 | 2 | 3 | 4 |  |
| Agaricomycetes sp.1 | LC483891 | 465 | GQ268624.1 | 98 | SH1555114.08FU | 0 | 1 | 0 | 0 | - |
| *Amanita* sp1 | LC483889 | 662 | KT072736.1 | 86 | SH1558502.08FU | 1 | 0 | 0 | 0 | T |
| *Amanita* sp*2* | LC483890 | 625 | GU222314.1 | 86 | SH1558502.08FU | 0 | 1 | 0 | 0 | T |
| *Amanita* sp3 | LC483913 | 389 | GQ268550.1 | 98 | SH1558521.08FU | 0 | 1 | 0 | 0 | T |
| *Amanita* sp4 | LC483914 | 585 | KP071209.1 | 98 | SH1558430.08FU | 0 | 0 | 1 | 0 | T |
| Atheliaceae sp1 | LC483891 | 665 | AB831856.1 | 95 | SH1548380.08FU | 4 | 0 | 1 | 0 | T,E |
| Atheliaceae sp2 | LC483915 | 571 | AB831856.1 | 86 | SH1548380.08FU | 0 | 0 | 1 | 0 | T |
| Atheliaceae sp3 | LC483916 | 571 | AB831856.1 | 86 | SH1548380.08FU | 0 | 0 | 1 | 0 | T |
| *Austroboletus* sp1 | LC483917 | 621 | KP242215.1 | 86 | SH1650532.08FU | 1 | 0 | 0 | 0 | T |
| *Austroboletu*s sp2 | LC483918 | 629 | KP242215.1 | 87 | SH1650532.08FU | 0 | 1 | 0 | 0 | T |
| *Boletaceae* sp1 | LC483919 | 687 | GQ268589.1 | 99 | SH1652208.08FU | 0 | 1 | 0 | 0 | T |
| Boletales 1 | LC483892 | 564 | GQ268583.1 | 86 | SH1657467.08FU | 2 | 0 | 0 | 0 | T |
| *Boletellus* sp1 | LC483920 | 179 | AB989020.1 | 86 | SH1565936.08FU | 0 | 0 | 0 | 1 | - |
| Clavariaceae 1 | LC483895 | 649 | LC098737.1 | 82 | SH1653968.08FU | 1 | 0 | 1 | 0 | T |
| *Clavulina* sp1 | LC483922 | 519 | HE601872.1 | 84 | SH1634947.08FU | 0 | 1 | 0 | 0 | - |
| Clavulinaceae sp1 | LC483896 | 833 | FJ196908.1 | 87 | SH1606333.08FU | 0 | 2 | 0 | 3 | T,E |
| Clavulinaceae sp2 | LC483923 | 624 | KJ786673.1 | 92 | SH1566766.08FU | 0 | 0 | 0 | 1 | - |
| Clavulinaceae sp3 | LC483924 | 621 | KP889716.1 | 79 | SH1606319.08FU | 0 | 0 | 0 | 1 | - |
| Clavulinaceae sp4 | LC483925 | 621 | KJ786673.1 | 89 | SH1566766.08FU | 0 | 0 | 0 | 1 | - |
| *Cenococcum geophilum 1* | LC483893 | 657 | DQ474356.1 | 95 | SH1639590.08FU | 5 | 1 | 8 | 0 | T,Q |
| *Cenococcum geophilum 2* | LC483894 | 623 | [DQ474343](https://www.ncbi.nlm.nih.gov/nucleotide/DQ474343.1?report=genbank&log$=nucltop&blast_rank=1&RID=6CX81YUJ014).1 | 95 | SH1230711.08FU | 0 | 1 | 0 | 0 | T |
| *Coltricia* sp1 | LC483926 | 534 | EF619668.1 | 87 | SH1571442.08FU | 0 | 1 | 0 | 0 | - |
| *Coltriciella* sp1 | LC483897 | 628 | KM594923.1 | 92 | SH1539199.08FU | 0 | 1 | 0 | 0 | E |
| *Coltriciella* sp2 | LC483898 | 624 | KM594897.1 | 87 | SH1539201.08FU | 0 | 1 | 0 | 1 | - |
| *Coltriciella* sp3 | LC483927 | 540 | AM412254.1 | 81 | SH1610842.08FU | 0 | 1 | 0 | 0 | T |
| *Coltriciella* sp4 | LC483928 | 569 | KJ540931.1 | 88 | SH1539198.08FU | 0 | 0 | 0 | 1 | T |
| Cortinariaceae 1 | LC483899 | 636 | GQ268604.1 | 98 | SH1504609.08FU | 0 | 2 | 0 | 0 | T,S |
| *Cortinarius* sp1 | LC483900 | 852 | EU821685.1 | 89 | SH1503735.08FU | 0 | 0 | 0 | 1 | T |
| *Cortinarius* sp2 | LC483901 | 613 | NR_119793.1 | 87 | SH1504970.08FU | 0 | 1 | 0 | 0 | T |
| *Cortinarius* sp3 | LC483902 | 808 | GQ159763.1 | 90 | SH1504486.08FU | 1 | 0 | 0 | 0 | T |
| *Craterellus* sp1 | LC483929 | 621 | AB922891.1 | 87 | SH1658813.08FU | 0 | 1 | 0 | 0 | - |
| *Craterellus* sp2 | LC483930 | 624 | AB922891.1 | 85 | SH1658813.08FU | 0 | 1 | 0 | 0 | T |
| *Craterellus* sp3 | LC483931 | 658 | AB922891.1 | 88 | SH1658813.08FU | 0 | 1 | 0 | 0 | - |
| *Elaphomyces* sp1 | LC483903 | 784 | KM595020.1 | 95 | SH1517916.08FU | 1 | 1 | 0 | 0 | T |
| *Heimioporus* sp | LC483904 | 631 | KR061493.1 | 99 | SH1521978.08FU | 0 | 1 | 0 | 0 | T |
| *Laccaria* sp | LC483905 | 787 | JX316722.1 | 93 | SH1553007.08FU | 1 | 0 | 0 | 0 | T |
| *Lactarius* sp1 | LC483932 | 750 | AJ534901.1 | 82 | SH1519123.08FU | 0 | 1 | 0 | 0 | Q |
| *Russula* sp1 | LC483906 | 791 | KU141303.1 | 90 | SH1569742.08FU | 4 | 0 | 1 | 0 | T,Q |
| *Russula* sp2 | LC483907 | 631 | KF245487.1 | 92 | SH1513803.08FU | 4 | 0 | 0 | 0 | T |
| *Russula* sp3 | LC483908 | 656 | KR135351.1 | 89 | SH1567172.08FU | 1 | 1 | 1 | 0 | E |
| *Russula* sp4 | LC483933 | 845 | GQ268658.1 | 96 | SH1564065.08FU | 0 | 1 | 0 | 0 | T |
| *Russula* sp5 | LC483934 | 407 | AB769908.1 | 96 | SH1564065.08FU | 0 | 1 | 0 | 0 | - |
| *Russula* sp6 | LC483935 | 621 | AB973714.1 | 96 | SH1509164.08FU | 0 | 0 | 0 | 1 | - |
| *Sebacina* sp | LC483909 | 752 | JQ347196.1 | 91 | SH1572381.08FU | 0 | 0 | 2 | 0 | T |
| Thelephoraceae sp1 | LC483910 | 926 | AB629001.1 | 93 | SH1642927.08FU | 6 | 0 | 1 | 0 | T |
| Thelephoraceae sp2 | LC483911 | 864 | AM412281.1 | 93 | SH1528926.08FU | 4 | 0 | 2 | 0 | T |
| Thelephoraceae sp3 | LC483936 | 611 | AB634262.1 | 93 | SH1642921.08FU | 1 | 0 | 0 | 0 | T |
| Thelephoraceae sp4 | LC483937 | 621 | GQ268666.1 | 75 | SH1642921.08FU | 1 | 0 | 0 | 0 | T |
| Thelephoraceae sp5 | LC483938 | 621 | AB629001.1 | 73 | SH1642921.08FU | 1 | 0 | 0 | 0 | T |
| Thelephoraceae sp6 | LC483939 | 611 | AM412281.1 | 86 | SH1528926.08FU | 1 | 0 | 0 | 0 | - |
| Thelephoraceae sp7 | LC483940 | 611 | AM412281.1 | 87 | SH1528926.08FU | 1 | 0 | 0 | 0 | - |
| Thelephoraceae sp8 | LC483941 | 596 | AM412281.1 | 88 | SH1528926.08FU | 0 | 0 | 1 | 0 | - |
| Thelephoraceae sp9 | LC483942 | 598 | AM412281.1 | 88 | SH1528926.08FU | 0 | 0 | 1 | 0 | T |
| Thelephoraceae sp10 | LC483943 | 621 | AM412281.1 | 87 | SH1528926.08FU | 0 | 0 | 1 | 0 | T |
| Thelephoraceae sp11 | LC483944 | 228 | JX456716.1 | 90 | SH1642922.08FU | 0 | 0 | 1 | 0 | T |
| *Xerocomus* sp1 | LC483921 | 761 | DQ822794.1 | 87 | SH1644963.08FU | 0 | 1 | 0 | 0 | Q |

*T, *Tristaniopsis*; S, *Shorea*; Q, *Quercus*; E, *Eucalyptus*
